# Current Role of Artificial Intelligence in the Management of Esophageal Cancer

**DOI:** 10.3390/jcm14061845

**Published:** 2025-03-09

**Authors:** Evgenia Mela, Dimitrios Tsapralis, Dimitrios Papaconstantinou, Panagiotis Sakarellos, Chrysovalantis Vergadis, Michail E. Klontzas, Ioannis Rouvelas, Antonios Tzortzakakis, Dimitrios Schizas

**Affiliations:** 1 First Department of Surgery, National and Kapodistrian University of Athens, Laikon General Hospital, 11527 Athens, Greece; panagiotissakarellos@gmail.com; 2 Department of Surgery, General Hospital of Ierapetra, 72200 Ierapetra, Greece; tsapralisd@yahoo.gr; 3 Third Department of Surgery, National and Kapodistrian University of Athens, Attikon University Hospital, 12462 Athens, Greece; dimpapa7@hotmail.com; 4 Department of Radiology, Laikon General Hospital, 11527 Athens, Greece; valvergadis@yahoo.gr; 5 Department for Clinical Science, Intervention and Technology (CLINTEC), Division of Radiology, Karolinska Institutet, 14152 Stockholm, Sweden; miklontzas@gmail.com (M.E.K.); antonios.tzortzakakis@ki.se (A.T.); 6 Department of Medical Imaging, University Hospital of Heraklion, 71500 Crete, Greece; 7 Computational BioMedicine Laboratory, Institute of Computer Science, Foundation for Research and Technology (FORTH), 71500 Heraklion, Greece; 8 Department of Radiology, School of Medicine, University of Crete, Voutes Campus, 70013 Heraklion, Greece; 9 Department of Clinical Science, Intervention and Technology (CLINTEC), Division of Surgery and Oncology, Karolinska Institutet, 14152 Stockholm, Sweden; ioannis.rouvelas@ki.se; 10 Department of Upper Abdominal Diseases, Karolinska University Hospital, Huddinge, 14152 Stockholm, Sweden; 11 Medical Radiation Physics and Nuclear Medicine, Section for Nuclear Medicine, Karolinska University Hospital, Huddinge, 14152 Stockholm, Sweden

**Keywords:** artificial intelligence, esophageal cancer, diagnosis, treatment, prognosis

## Abstract

**Background/Objectives**: Esophageal cancer (EC) represents a major global contributor to cancer-related mortality. The advent of artificial intelligence (AI), including machine learning, deep learning, and radiomics, holds promise for enhancing treatment decisions and predicting outcomes. The aim of this review is to present an overview of the current landscape and future perspectives of AI in the management of EC. **Methods**: A literature search was performed on MEDLINE using the following keywords: “Artificial Intelligence”, “Esophageal cancer”, “Barrett’s esophagus”, “Esophageal Adenocarcinoma”, and “Esophageal Squamous cell carcinoma”. All titles and abstracts were screened; the results included 41 studies. **Results**: Over the past five years, the number of studies focusing on the application of AI to the treatment and prognosis of EC has surged, leveraging increasingly larger datasets with external validation. The simultaneous incorporation in AI models of clinical factors and features from several imaging modalities displays improved predictive performance, which may enhance patient outcomes, based on direct personalized therapeutic options. However, clinicians and researchers must address existing limitations, conduct randomized controlled trials, and consider the ethical and legal aspects that arise to establish AI as a standard decision-support tool. **Conclusions**: AI applications may result in substantial advances in EC management, heralding a new era. Considering the complexity of EC as a clinical entity, the evolving potential of AI is anticipated to ameliorate patients’ quality of life and survival rates.

## 1. Introduction

Esophageal cancer represents the eighth-most prevalent malignancy worldwide and constitutes a leading cause of cancer-related mortality [1,2]. The global burden of esophageal cancer has grown more than sixfold, attributed to the world’s aging population along with the rising prevalence of related risk factors, the most prominent being tobacco and alcohol use alongside obesity [2]. By 2030, esophageal adenocarcinoma is expected to experience a significant rise in incidence, while esophageal squamous cell carcinoma is projected to decline steadily [3]. Associated with a dismal prognosis, the reported 5-year survival rate is 22%, which declines to 6% in the occurrence of distant metastases [4]. EC is estimated to decrease the absolute years of life by 7.8 (95% confidence interval: 2.3–12.7) [5].

The advent of AI, which simulates human cognitive abilities, has revolutionized numerous disciplines. AI constitutes an appealing approach to medical management mainly by employing two major tools: traditional machine learning (ML) and deep learning (DL) [6]. The implementation of ML and DL techniques lessens the prospect of human error. ML is the discipline in which models are trained through automated learning using expert-provided data to identify specified patterns. DL, a subset of ML, utilizes multilayered neural networks and larger datasets and simulates a more complex form of learning that enhances accuracy and outcomes over time [7]. Meanwhile, radiomics offers a quantitative perspective on imaging data, permitting the recognition of fine patterns corresponding to the tumor’s biological behavior and heterogeneity that are imperceptible through conventional means [8].

EC remains a significant global health concern, affecting both its diagnosis and management [1,9]. The emergence of AI and its application for diagnostic, therapeutic, and prognostic purposes presents an opportunity to improve the poor prognosis associated with EC [9]. AI has been utilized across the entire spectrum of EC management, particularly in diagnosis. In this regard, hyperspectral and multispectral imaging techniques, which constitute AI-based diagnostic imaging techniques, have been employed to enhance EC detection rates with encouraging outcomes [10,11,12]. While AI has been primarily employed in the identification of early precursor lesions and to enhance prognosis, notable advancements have also been made in its role in patient care, including treatment optimization and survival prediction [9]. Given the scarcity of non-invasive biomarkers, there is a significant reliance on radiological imaging during treatment planning and assessment, wherein AI could serve a pivotal role [8].

The purpose of this review is to critically evaluate and consolidate the existing literature concerning the use of AI for optimizing treatment decisions and their implementation in the prognosis prediction of EC. To our knowledge, this is the first narrative review that focuses exclusively on the benefits of AI in the management of EC, aiming to provide an overview of its current and future applications.

## 2. Materials and Methods

We performed a literature search of MEDLINE from its start date up to June 2024. The search strategy included the following terms: “Artificial Intelligence”, “Esophageal cancer”, “Barrett’s esophagus”, “Esophageal Adenocarcinoma” and “Esophageal Squamous cell carcinoma”. Eligibility criteria included randomized controlled trials, non-randomized cohort or prospective studies, and retrospective studies, which were published in the English language and referred to applications of AI in treatment decisions, planning, surgical treatment, and prognostic prediction of EC. Articles without an available full text and irrelevant or unavailable data were excluded. The exclusion criteria also included conference abstracts, preprints, and non-English studies. There were no particular inclusion or exclusion criteria defined by AI model validation methods or sample size.

**Figure 1:**
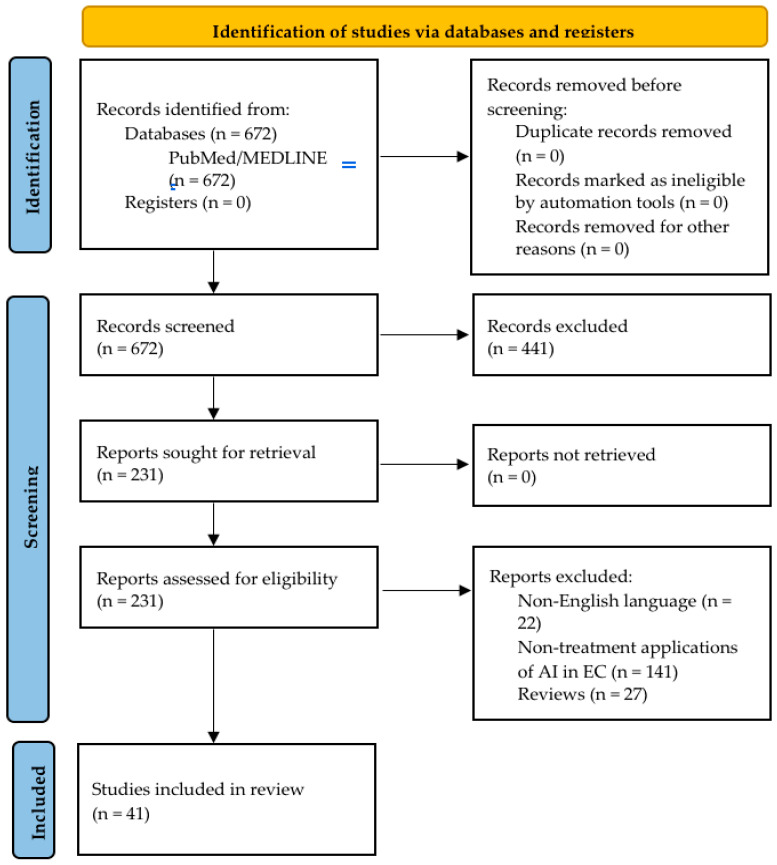
PRISMA flowchart of study selection.

The titles and abstracts of all articles were initially independently screened by two researchers (E.M and D.P.), and any ensuing conflicts were addressed by a third researcher (D.T.). Full-text articles were subsequently reviewed for data extraction. Ultimately, 41 studies were included (Figure 1), and considering the heterogeneity of the study pool, a narrative presentation was chosen.

## 3. AI Applications in EC Treatment and Prognosis Prediction

### 3.1. AI in Treatment Decision and Planning

AI has the potential to revolutionize the current multidisciplinary treatment planning for EC by further optimizing effective time management and ensuring consistency in the practices of multidisciplinary tumor boards. In 2023, British experts introduced a multinomial logistic regression algorithm aiming at replicating the decision-making of the upper gastrointestinal multidisciplinary boards, which was tested retrospectively. The findings, utilizing a single-center database, demonstrated good performance, with a mean area under the curve (AUC) of 0.793. Notably, age was identified as the most significant variable affecting predictive accuracy [13]. Additionally, to facilitate multidisciplinary team decisions, Zahedi et al. developed a multilayer neural network incorporating particle swarm optimization and an error back propagation program to calculate the optimal chemotherapy dosage. Particle swarm optimization is a bio-inspired program that simulates the collective behavior of social creatures, such as birds. It is formed by a swarm of particles, each reflecting a solution to an optimization issue, which collectively modify their velocity and position to attain the global optimal state. Simultaneously, error back propagation is a supervised training approach for artificial neural networks (ANNs) that shifts the weights of the network in response to the error function. The model was tested retrospectively and achieved a reported accuracy of 77.3% [14].

Supplementary treatment-planning algorithms for EC have likewise been developed for radiotherapy. In 2021, Barragán-Montero et al. emphasized the importance of a large, high-quality dataset for constructing DL models for the dosage prediction of intensity-modulated radiotherapy (IMRT) [15]. Meanwhile, a key aspect of radiation planning is clinical target volume (CTV) segmentation, which demands time and relies on the physician’s expertise and experience [16]. The first study on CTV auto-segmentation, employing CT scans for stage I and II esophageal cancer after surgical resection, utilized a deep dilated convolutional U-network (DDUnet) model architecture for image segmentation. DDUnet delivers a more comprehensive image segmentation compared to the traditional Unet by retrieving and maintaining contextual data from the provided images. The study documented an 86,7% dice similarity coefficient (DSC) value and a total time of 25 s per patient [16]. Similarly, Jin et al. focused on convolutional neural network (CNN)-based models for gross tumor volume contour delineation and found that their proposed algorithm, VUMix-Net, achieved superior quantitative and qualitative performance, comparable to that of human experts, particularly for the middle third of the esophagus [17]. Subsequently, with the intent of maximizing time and workload efficiency, alongside the accuracy of radiation dosage allocation, an asymmetrical 3D ResNet DL model was proposed for patients receiving a range of prescribed doses. The model projected dose distribution with a mean relative error of 2% and an average DSC for isodose volumes of 0.93. In addition, it was demonstrated that the model’s incorporation enhanced clinical workflow efficacy by elevating the accuracy of clinical decisions for conventional or dose-reduced radiotherapy, from 95.24% to 100% for physicians, by significantly decreasing planning time by 61.57% for junior and 52.47% for senior dosimetrists, along with reducing the average dosage variance by 78.64% among the two [18].

With encouraging findings and the potential of personalized treatment plans, numerous predictive models have been generated for estimating radiation-derived complications. Zhu et al. developed an ML approach for predicting radiotherapy-related immunotoxicity in EC patients undergoing proton and photon treatment. The proposed model displayed an AUC of 0.783 and an accuracy of 0.717, whereas parameters such as age, absolute lymphocyte count, and cardiopulmonary dosage were found to influence lymphopenia risk when shifting to proton therapy [19]. Yet, Chinese researchers created another model, HybridNet, incorporating pulmonary dose distribution and clinical features to predict the occurrence of radiation pneumonitis. HybridNet exhibited an accuracy of 0.85 and an AUC of 0.91, highlighting its potential as a future decision-support tool in order to simplify clinical workflow and optimize treatment quality [20].

### 3.2. AI in EC Surgical Treatment

Esophagectomy remains the cornerstone treatment for localized EC. Despite the current applications of AI in surgery being limited, substantial research has been conducted. In the study of Sato et al., a DL algorithm for image analysis was developed to accurately detect the position of recurrent laryngeal nerves intraoperatively. This software surpassed the average performance of general surgeons’ and nearly matched that of upper gastrointestinal surgeons [21]. With the future prospect of aiding in the intraoperative identification of tumor margins and the achievement of a higher percentage of R0 resection, Maktabi et al. introduced a hyperspectral imaging algorithm, which identified malignant tissue with a sensitivity of 63% and a specificity of 69%, within a timeframe of less than a second [22]. Nonetheless, the inquiry for future research persists regarding the potential of AI to facilitate the achievement of greater R0 rates. Likewise, AI algorithms have been devised to automatically identify endoscopic or surgical phases, enabling potential continuous monitoring and enhancing team performance. Particularly, the model applied in robot-assisted esophagectomy displayed an accuracy rate of 84%, whereas the initial model deployed for endoscopic submucosal dissection, which is considered the mainstay of treatment for superficial tumors, indicated an overall accuracy of 90% [23,24]. This superior performance in endoscopic settings can be attributed to the uniformity and the minimal anatomic variations encountered during surgical procedures [23].

Considering that esophagectomy is a procedure carrying an increased risk of complications, Jung et al. constructed a neural network to predict their occurrence. The results indicated a poor overall accuracy of 0.688 and an AUC of 0.672 for complications equal to or greater than IIIa, according to the Clavien–Dindo classification. In this context, the neural network outperformed the Cologne risk score, which is an established clinical risk calculator, in terms of accuracy for predicting Clavien–Dindo IIIa or higher complications (accuracy 0.688 versus 0.510, respectively) and surgical complications (accuracy 0.667 versus 0.624, respectively) [25]. Similarly, van Kooten et al. conducted a comparative study to assess the advantages of ML in conjunction with linear regression algorithms in forecasting anastomotic leakage and respiratory sequelae following upper gastrointestinal tract oncologic surgery. The researchers found that the linear regression model accomplished the highest overall prediction accuracy for esophageal surgery, followed by neural networks for estimating anastomotic leak probability [26]. A novel ML hybrid algorithm has been recently introduced, integrating preoperative CT and clinical variables in order to accurately estimate the probability of an anastomotic leak. The reported performance was an AUC of 79.2%, facilitating the prompt identification and treatment of an anastomotic leak, even in cases with minimal clinical suspicion [27]. Algorithms have also been generated to estimate early postoperative re-admissions within 30 days, and it has been demonstrated that ML models can disclose associations that are imperceptible by traditional regression algorithms and discover independent predictive variables for complications or re-admissions, hence enhancing perioperative treatment and recovery [26,27,28]. Table 1 summarizes the results of AI applications in surgical treatment.

### 3.3. AI in Treatment Response Prediction

Although preoperative assessment and prediction of treatment response are crucial for guiding individualized therapeutic decisions, this remains a challenge in clinical practice. Despite the potential confounding effects, involving differences in neoadjuvant treatment regimens amongst different protocols, numerous studies have established that imaging can serve as a prognostic indicator of treatment responsiveness. In 2020, Hu et al. demonstrated that incorporating both peritumoral and intratumoral radiomic features enhances predictive efficacy for pathological complete response (pCR) following neoadjuvant treatment in esophageal squamous cell carcinoma (ESCC) [29]. Subsequently, a comparative analysis was conducted between radiomics, CNNs, and clinical models, which reported that the optimal model integrating features by ResNet50, which is a CNN model for image classification, displaying an AUC of 0.805 and with all radiological algorithms outperforming the conventional clinical models [30]. However, a subsequent study indicated that the integration of clinical data with radiomics enhances performance, as demonstrated by a reported AUC of 0.891, as opposed to 0.817 for the radiomics-only model, in predicting the pCR of primary ESCC lesions [31]. In contrast to these retrospective studies, the multicenter study by Li et al. established an advanced three-dimensional prediction algorithm that demonstrated a 100% positive predictive value and an AUC of 0.833 in identifying non-responders to neoadjuvant chemoradiotherapy for locally advanced ESCC [32]. Additionally, a CT radiomics model was developed to estimate responses to immunotherapy and chemotherapy in patients with advanced ESCC. The study evaluated two-dimensional and three-dimensional CT radiomics models, which were established on 2D regions of interest (ROIs) at the level of greatest transverse tumor diameter and 3D ROIs derived by the entire tumor volume, respectively. The two-dimensional corrected model outperformed the three-dimensional model, yielding an accuracy of 79.6% in the validation cohort [33]. While the Response Evaluation Criteria in Solid Tumors (RECIST) serve as an established method for assessing residual disease in solid tumors, they exhibit limitations in EC due to indistinct margins or fibrotic tissue following neoadjuvant treatment, and there have been no direct comparisons in terms of performance among RECIST and neural networks for EC [34].

In the context of radiotherapy response, the absence of biomarkers precludes the individualization of treatment plans. To address this, researchers utilized the pre-trained ResNet50 for predicting radiotherapy response, achieving an AUC value of 0.732 in external validation [35]. In addition, Jin et al. demonstrated that merging radiomic and dosimetric variables enhances prediction accuracy and efficiency. Recently a dose map-guided AI model was introduced for estimating pCR, reporting an impressive AUC of 0.928 [36,37].

Although the reduction in tumor metabolic rate, as evaluated by standardized uptake value (SUV) within the second week of neoadjuvant treatment using 18F-FDG positron emission tomography (18F-FDG PET), has been recognized in the global literature as having predictive significance, its sensitivity and specificity remain below 70% [38,39]. Ypsilantis et al. were the first to develop a three-slice CNN, which employed PET scan data to predict response to neoadjuvant chemotherapy, with an accuracy of 73.4% and sensitivity and specificity exceeding 80% [40]. Furthermore, the combination of variables from PET scan and CT imaging delivers superior results compared to each modality individually, with a reported AUC of 0.87 [41]. Likewise, Qi et al., who first conducted a prospective study combining PET, CT, and clinical features, concluded that incorporating clinical data optimizes the performance of predictive models for ESCC patients undergoing neoadjuvant chemoradiotherapy and immunotherapy. Therefore, the multimodal support vector machine ML model significantly outperformed the ones utilizing only CT or PET variables in the testing dataset (AUC = 0.852, 95% CI: 0.824–0.876) [42].

Finally, in addition to imaging modalities, there have been advancements in the development of computational models making use of endoscopic images for predicting response following neoadjuvant therapy. The initial assessment of a deep neural network endoscopy-based algorithm for predicting pCR using pretreatment images in locally advanced ESCC was conducted by Kawahara et al. in 2022 [43]. An accuracy rate of 64% was recorded, which subsequently rose to 80.6% when imaging filters were employed [43]. Similarly, Matsuda et al. constructed an AI-based predictive model for pCR using pretreatment endoscopy images for ESCC patients. Their findings indicated a moderate degree of accuracy, surpassing that of endoscopists [44]. The researchers also created an AI-guided endoscopic response assessment model for ESCC patients following neoadjuvant treatment, achieving comparable or superior results compared to the average performance of endoscopists [45]. Table 2 summarizes the outcomes of the aforementioned studies.

### 3.4. AI in EC Prognosis

One of the most significant applications of AI is the prediction of disease prognosis. The traditional primary tumor, lymph nodes, and distant metastasis (TNM) staging system has long been utilized as the main indicator for patient outcomes [46]. However, in the fundamental study of Sato et al. ANNs were implemented for clinicopathologic, biologic, and genetic variables and significantly outperformed the TNM classification system in predicting 1- and 5-year survival rates (*p* < 0.0001) [47]. In support of the use and the superiority of ANNs were also Modifi et al. for survival estimates following surgical resection. In particular, the ANNs displayed a statistically significant superiority in accuracy compared to the TNM classification system for 1-year disease-free survival (DFS), 88% versus 71.6% (*p* < 0.01), and for 3 years, at 91.5% versus 74.7% (*p* < 0.05) [48].

In addition to ANNs, the emergence of radiomics and their extensive application in the quantitative interpretation of imaging data has resulted in their integration into predictive survival models. While Larue et al. underlined that CT radiomics models achieve greater prognostic performance than the clinical models due to the additional features defining tumor heterogeneity, Luo et al. demonstrated that the combined model integrating radiomics and clinical features was superior for predicting local progression-free survival [49,50]. In this context, Cui et al. developed a composite ML model to predict progression-free survival (PFS) and overall survival (OS) in non-surgical ESCC patients and concluded that incorporating clinical data improves predictive efficacy, reporting an AUC of 0.856 and 0.742 for PFS and OS respectively [51]. Furthermore, a DL radiomics model has been assessed in conjunction with handcrafted features radiomics model, such as the tumor’s shape and texture. The DL radiomics model exhibited an advantage with regard to Harrel’s concordance index and AUC, correlating with 3-year OS [52]. In 2023, Zhang H et al. established and validated a new DL staging system for patients with ESCC using a database of 6020 patients. The novel system displayed a remarkable capacity for differentiation regarding 3- and 5-year OS rates [53]. Subsequently, Zhang K et al. sought to forecast the OSs of patients with ESCC by using a Cox proportional hazards (CoxPH) model utilizing a substantial dataset and machine-learning techniques. The optimal model, which comprised 10 variables, demonstrated an AUC of 0.760, surpassing the risk model that incorporated the five most important variables, with an AUC of 0.745, and achieved superior distinctive ability compared to the AJCC eighth staging system [54].

Likewise, magnetic resonance imaging (MRI) has been utilized alongside models incorporating CT radiomics features, demonstrating remarkable consistency in tumor imaging. Combining MRI-based radiomics features with clinical parameters yielded good predictive ability for DFS and OS in ESCC patients [55].

Predictive models have also been created to assess survival and clinical effectiveness following immunotherapy. PD-L1 expression constitutes a prominent biomarker, despite its uncertain prognostic significance [56,57]. For this purpose, Chen et al. established a seven-immune gene model that exhibited an AUC performance of 0.825 at 1 year for the testing group, with the limitation of low efficiency at 3 years due to an insufficient number of patients with follow-up exceeding 3 years [58]. Furthermore, Li B et al. developed a DL pathomics system, named ESCC-PS, to estimate PFS following immunotherapy, with a concordance index of 0.806. This model outperformed PD-L1, indicating that ESCC-PS provides supplemental prognostic information [59]. Table 3 consolidates the findings for AI in EC prognosis prediction.

## 4. Limitations

Among the limitations of this review is its narrative methodology, which differs from a systematic analysis of the current literature. Consequently, it is inherently more susceptible to selection bias. In this context, this descriptive presentation may hinder generalizability due to the variability in AI model development, preprocessing techniques, and validation methods among the included studies. The lack of systematic analysis of bias in AI algorithms, data privacy concerns, and regulatory approval protocols also stems from the narrative methodology. Furthermore, bridging the gap between research and clinical practice requires the application of AI on larger datasets within the context of multicenter clinical trials and the direct comparison of AI models to conventional treatment and prognostic assessment. Finally, given that ESCC is the predominant histological type globally, particularly in Eastern Asian countries, the majority of the included papers originate from Asia, potentially affecting the generalizability of the findings.

## 5. Future Perspectives

The current landscape highlights the substantial potential of AI in treatment decision-making and outcome prediction for EC patients. Ongoing clinical trials and observational studies aim to address the challenges that must be overcome before AI can be integrated into clinical practice. Specifically, researchers in Singapore and China are working to develop and assess a clinical decision-assistance system for upper gastrointestinal tract malignancies. In particular, this clinical trial aims to develop and prospectively validate a clinical decision support system, along with comparing it with the recommendations of expert oncological teams in order to improve therapeutic decision making in upper gastrointestinal malignancies (NCT04675138). Concurrently, a prediction algorithm for symptoms and complications following chemotherapy is under development for several malignancies, including EC. The objective of this prospective observational study is to acquire disease-specific biomarkers from EC patients undergoing chemotherapy via wearable devices while concurrently measuring self-reported adverse events, emergency department visits, and chemotherapy completion rates through an application, to ultimately develop an AI-based complication prediction algorithm based solely on biomarkers (NCT05937477). Meanwhile, a research team from India is constructing an AI-based system to predict the pathological response of EC to neoadjuvant chemoradiotherapy and aims to minimize the ‘black box’ phenomenon, wherein the underlying rationale of a CNN remains obscure, while also demonstrating the biological coherence of the predictions, enhancing thus the transparency, comprehensibility, and interpretability of the AI models. This observational study recognizes the lack of transparency of CNNs and aims to address it by utilizing activation maps that highlight the critical areas of an image pertinent to a given prediction, thus illustrating the biological plausibility of the predictions (NCT04489368).

In this context, due to the scarcity of contemporary research in esophageal cancer that improves AI transparency and explainability, there is an imperative need for more studies focusing on interpretative methods to augment AI comprehensibility. Several interpretation tools, including SHAP, LIME, and Grad-CAM, have been introduced in the medical field to elucidate the decision-making process of DL models [60]. Furthermore, causal inference offers a novel approach for investigating causality as opposed to simple correlation, hence enhancing the reliability of AI prediction in the medical field [61]. The ethical and legal implications also arising from this ‘black box’ nature warrant further examination. These encompass issues of privacy, surveillance, bias, and the overarching philosophical controversy over the significance of human reasoning [62]. An additional major challenge is the lack of explicit accountability in instances of errors, privacy violations, and mishandling of big datasets. Therefore, to effectively leverage AI’s potential in EC management, it is essential to consider securing informed consent approval for data utilization, upholding safety, and AI explainability, minimizing algorithmic biases and safeguarding confidentiality of information [63]. Nevertheless, as AI is becoming a clinical decision-support tool, developers must integrate additional human behavioral and reasoning factors into the decision-making process and address the lack of direct comparisons among AI models and established surgical risk calculators, treatment responses, and prognostic evaluation methods to bridge the gap between theory and clinical practice.

## 6. Conclusions

In conclusion, the emergence of AI tends to revolutionize the management of EC patients. This review outlines the prospects of AI applications in the treatment and prognosis of EC. Numerous models have demonstrated performance that equals or exceeds that of healthcare specialists in treatment decision-making. However, despite the promising capabilities of AI and the encouraging results, further multicenter clinical trials must be conducted to eliminate current barriers and facilitate in the future its integration into routine clinical practice. Given the complex nature of EC, the implementation of these AI-driven approaches is expected to allow their regular use as decision-support systems, ultimately aiming to improve patients’ quality of life and survival rates.

## Figures and Tables

**Table 1:** Applications for surgical treatment.

Author	Year	Sample Size	Study Design	Objective	Results
Sato K et al. [21]	2022	3000 images from 20 thoracoscopic videos and 40 images from 8 thoracoscopic videos	Retrospective	Development of a DL-based AI image model for identifying the location of the recurrent laryngeal nerve and assess if it reduces the incidence of recurrent laryngeal nerve paralysis	The model had an average dice coefficient of 0.58. This was not significantly different from the group of specialized esophageal surgeons (*p* = 0.26) but was significantly superior to that of the group of certified general gastrointestinal surgeons (*p* = 0.019).
Maktabi M et al. [22]	2019	11 patients	Retrospective	Evaluation of intraoperative hyperspectral imaging for identifying the tumor margins intraoperatively	The support vector machines (SVM) algorithm has the best performance, with 63% sensitivity and 69% specificity for identifying cancerous tissue.
Furube T et al. [23]	2024	94 videos	Retrospective	Development of an AI-based automated ESD phase recognition system based on video images	The model displayed an overall accuracy of 90%, an average precision of 91%, and a recall of 90%.
Takeuchi M et al. [24]	2022	31 patients	Retrospective	Development of an AI-based automated surgical-phase recognition system for RAMIE	The model achieved an overall accuracy of 84%.
Jung JO et al. [25]	2023	864 patients	Retrospective	Development of ML-based methods for predicting Clavien–Dindo grade IIIa or greater complications following esophagectomy	The neural network achieved an overall accuracy of 68.8% and accuracies of 69.2% for medical complications, and 66.7% for surgical complications. The AUC of neural network was 0.672 for Clavien–Dindo grade IIIa or higher, 0.695 for medical complications, and 0.653 for surgical complications.
van Kooten RT et al. [26]	2022	4288 patients	Retrospective	Evaluation of the added value of ML methods for predicting postoperative complications following esophagectomy and development of a predictive model for anastomotic leakage and cardiopulmonary complications	The optimal model GLM displayed an AUC of 0.619 for anastomotic leakage and 0.644 for pulmonary complications.
Klontzas M et al. [27]	2024	471 patients	Retrospective	Development of an ML model combining CT and clinical variables for predicting anastomotic leakage following esophagectomy	The XGboost model achieved an AUC of 0.792, 77.46% specificity, 69.22% sensitivity, 48.39% positive predictive value, and 87.3% negative predictive value.
Bolourani S et al. [28]	2021	2037 patients	Retrospective	Development of ML-based prediction model for early readmissions within 30 days following esophagectomy	The ML model for clinical decision achieved a sensitivity of 71.7% and specificity of 51.4%, while the ML model for quality review achieved an accuracy of 84.8%, specificity of 98.7%, and sensitivity of 23%

DL: deep learning, AI: artificial intelligence, RAMIE: robot-assisted minimally invasive esophagectomy, ESD: endoscopic submucosal dissection, ML: machine learning.

**Table 2:** Treatment response prediction.

Author	Year	Sample Size	Study Design	Treatment	Objective	Results
Hu Y et al. [29]	2020	231	Retrospective	nCRT and surgery	Development and validation of CT-based models to identify pCR using intratumoral and peritumoral features in ESCC patients	The model combining 7 intratumoral and 6 peritumoral features displayed superior discriminative performance, with an AUC of 0.852 (95% CI, 0.753–0.951), accuracy of 84.3%, sensitivity of 90.3% and specificity of 79.5% in the test set.
Hu Y et al. [30]	2021	231	Retrospective	nCRT and surgery	Evaluation and validation of a CT-based model using DL features for predicting pCR to nCRT in ESCC patients	The model integrating features from ResNet50 achieved an AUC of 0.805 (95% CI, 0.696–0.913) and an accuracy of 77.1%, compared with 0.725 (95% CI, 0.605- 0.846) and 67.1% for the radiomics model in the test set.
Wang J et al. [31]	2023	112	Retrospective	nCRT and surgery	Development and validation of a radiomics feature-based ML model for predicting pCR to nCRT in ESCC patients	For pCR prediction models, the model combining rad-score and clinical features achieved an AUC of 0.891 (95% CI, 0.823–0.950), compared with 0.817 (95% CI, 0.732–0.896) for the radiomics model in the test set.
Li X et al. [32]	2021	306	Prospective	nCRT	Development and validation of a 3-dimensional DL radiomics model for predicting response to nCRT in ESCC patients	The model achieved an AUC of 0.897 (95% CI, 0.840–0.959) in the training cohort and 0.833 (95% CI, 0.654–1.000) in the validation cohort
Zhu Y et al. [33]	2021	64	Retrospective	Chemotherapy and PD-1 inhibitor	Development and validation of a radiomics model for evaluating treatment response to chemotherapy and immunotherapy in ESCC patients	The 2-dimensional corrected model achieved an AUC of 0.843 (95% CI, 0.736–0.950) in the training cohort and 0.914 (95% CI, 0.775–1.000) in the validation cohort, compared to the 3-dimensional corrected model with an AUC 0.658 (95% CI, 0.502–0.813) and 0.670 (95% CI, 0.511–0.849) respectively
Xie Y et al. [35]	2023	248	Retrospective	nCRT	Establishment and validation of an ANN-based radiomics model for predicting radiotherapy response in patients with stage III ESCC	The pretrained network ResNet50 displayed superior performance, with AUCs of 0.876, 0.802, and 0.732 in the training, internal validation, and external validation cohorts respectively
Jin X et al. [36]	2019	94	Retrospective	CRT	Evaluation of a model combining CT radiomics and dosimetric features for predicting response to CRT in esophageal cancer patients	The XGBoost plus principal component analysis model combining radiomics features with dosimetric parameters achieved a prediction accuracy of 70.8% and an AUC of 0.689 compared to the radiomics model with an accuracy of 62.5% and AUC of 0.412
Yap WK et al. [37]	2023	80	Retrospective	nCRT and surgery	Development of radiotherapy dose map-guided DL model for predicting ypCR to nCRT in ESCC patients	The HRNetV2p model with dose contextual representations achieved superior performance, with an AUC of 0.928 (95% CI, 0.884–0.972)
Ypsilantis PP et al. [40]	2015	107	Prospective	nCT	Evaluation of the predictive ability of an ML algorithm (3S-CNN) for representing esophageal cancer’s metabolic profile using 18F-FDG PET imaging	The 3S-CNN achieved superior performance with an average 80.7% sensitivity, 81.6% specificity, and 73.4% accuracy in predicting non-responders
Rishi A et al. [41]	2020	68	Retrospective	nCRT and surgery	Development and validation of a combined CT and PET/CT radiomics model for predicting pCR to nCRT in patients with advanced esophageal cancer	The combined PET/CT radiomics model achieved improved predictive performance with an AUC of 0.87 compared to CT- or PET-only models with AUCs of 0.73 and 0.66 respectively
Qi WX et al. [42]	2024	126	Prospective	nCRT, anti-PD1 inhibitors and surgery	Evaluation of an integrated multimodal radiomics with ML model for predicting pCR to nCRT and anti-PD1 in ESCC patients	Support vector machine ML trained on CT, PET, and clinical features achieved superior performance compared to CT and PET-only models with an AUC of 0.997 in the training set and 0.852 in the testing set
Kawahara D et al. [43]	2022	98	Retrospective	nCRT and surgery	Proposal of a DL-based model using endoscopic images for predicting pCR to nCRT in ESCC patients	The model achieved 64% accuracy, 80.3% sensitivity, 36.3% specificity, and 0.58 AUC in the testing set compared to 80.6%, 79.8%, 80.6%, and 0.83 with wavelet filter, respectively
Matsuda S et al. [44]	2023	123	Retrospective	nCRT and surgery	Development of a deep neural network using endoscopic images for identifying pCR to nCT preoperatively in ESCC patients	In 20 models, the median sensitivity, specificity, positive predictive value, negative predictive value, and accuracy for endoscopic response evaluation were 60%, 81%, 77%, 67%, and 70% compared to 43%, 90%, 85%, 65%, and 66% for endoscopists respectively
Matsuda S et al. [45]	2023	193	Retrospective	nCRT and surgery	Development of a deep neural network for evaluating endoscopic response to nCT in ESCC patient development of a deep neural network for evaluating endoscopic response to nCT in ESCC patients	In 10 models the median sensitivity, specificity, positive predictive value, and negative predictive value were 60%, 100%, 100%, and 71% compared to 80%, 80%, 81%, and 81% for endoscopists respectively

nCRT: neoadjuvant chemoradiotherapy, nCT: neoadjuvant chemotherapy, DL: deep learning, ML: machine learning, ANN: artificial neural network, pCR: pathological complete response.

**Table 3:** Prognosis prediction.

Author	Year	Sample Size	Study Design	Objective	Results
Sato F et al. [47]	2005	418	Retrospective	Development of an ANN model for predicting 1- and 5-year survival for esophageal cancer patients	The optimal ANN models for predicting 1- and 5-year survival consisted of 65 variables (AUR = 0.883) and 60 variables (AUR = 0.884), respectively, and outperformed the TNM solely based model (*p* < 0.0001).
Modifi R et al. [48]	2006	216	Retrospective	Evaluation of the performance of an ANN model for predicting 1- and 3-year disease-free survival for esophageal and EGJ carcinoma patients	The accuracy, sensitivity, and specificity of the ANN for predicting survival at 1 year was 88%, 92.3%, and 84.5%, and at 3 years was 91.5%, 94.6%, and 88%, and was statistically significantly superior to TNM classification system.
Larue RTHM et al. [49]	2018	239	Retrospective	Training and validation of two models using radiomics or clinical variables for predicting 3-year overall survival for esophageal cancer patients following nCRT.	The radiomics-based RF model achieved superior prognostic performance for 3-year overall survival with an AUC of 0.69 and 0.61 in the training and validation dataset compared to 0.63 and 0.62 for the clinical feature-based model, respectively.
Luo HS et al. [50]	2021	221	Retrospective	Development and validation of a radiomics and clinical feature-based model for predicting local progression-free survival for ESCC patients following concurrent CRT	The C-index of the prediction model was 0.745 (95% CI, 0.770–0.790) in the training cohort and 0.723 (95% CI, 0.654–0.791) in the validation cohort.
Cui Y et al. [51]	2022	204	RCT	Development of ML models for predicting progression-free survival and overall survival for ESCC patients	Combined models using clinical and radiomics features achieved over 70% accuracy, an AUC of 0.833, and a C-index of 0.79 for predicting PFS and 0.768 and 0.71 for predicting OS in the test cohort.
Wang J et al. [52]	2022	154	Retrospective	Development and validation of a DL radiomics-based model for predicting 3-year overall survival for esophageal cancer patients	The deep-learning radiomics model achieved an AUC of 0.984 and a C-index of 0.76 in the training cohort and 0.942 and 0.784 in the validation cohort for predicting 3-year OS, respectively.
Zhang H et al. [53]	2023	6020	Retrospective	Development and validation of a DL model for predicting overall survival and a novel staging system for ESCC patients	The deep-learning model achieved superior performance than the traditional nomogram for predicting OS with C-index 0.732 [95% CI 0.714–0.750] and 0.671 [95% CI 0.647–0.695], respectively. The model in the test cohort displayed an AUC of 0.805 at 3-year OS and 0.825 at 5-year OS
Zhang K et al. [54]	2023	2441	Retrospective	Development of an ML survival prediction model using 6 different ML g approaches for ESCC patients	The ML-extended CoxPH model achieved superior discriminative performance, with AUCs for 1-, 3-, and 5-year overall survival of 0.760, 0.735, and 0.746 in the training cohort and 0.725, 0.720, and 0.752 in the validation cohort, respectively.
Chu F et al. [55]	2022	434	RCT	Development and validation of an MRI-based radiomics model for predicting disease-free survival and overall survival in ESCC patients	The combined model based on 7 radiomics features and clinical features achieved the best performance, with a C-index of 0.730 in the training cohort and 0.712 in the validation cohort for OS and 0.714 and 0.729 for DFS respectively.
Chen H et al. [58]	2020	159	Retrospective	Development and validation of a 7 immune gene model for predicting prognosis of esophageal cancer	The model achieved an AUC of 0.825 for 1 year and 0.596 for 3 years in the test set.
Li B et al. [59]	2024	163	Retrospective	Assessment of a DL-based pathomics signature for predicting clinical benefits of immunotherapy in ESCC patients	The ESCC-PS achieved an accuracy of 84.5% in the validation cohort and the combined model based on ESCC-PS and PD-L1 expression displayed an AUC of 0.904 and a C-index of 0.814 for a 6-month PFS.

RCT: randomized controlled trial, nCRT: neoadjuvant chemoradiotherapy, DL: deep learning, ML: machine learning, ANN: artificial neural network.

## Abbreviations

The following abbreviations are used in this manuscript:

EC	Esophageal cancer
AI	Artificial Intelligence
ML	Machine learning
DL	Deep learning
AUC	Area under the curve
ANN	Artificial neural network
CTV	Clinical target volume
CNN	Convolutional neural network
pCR	Pathological complete response
ESCC	Esophageal squamous cell carcinoma
SUV	Standardized uptake value
PET	Positron emission tomography
PFS	Progression-free survival
OS	Overall survival
MRI	Magnetic resonance imaging
DFS	Disease-free survival
